# Epigenetic therapies: novel strategies for improving outcomes in myeloid malignancies

**DOI:** 10.1186/1868-7083-5-S1-S12

**Published:** 2013-08-19

**Authors:** Charles Craddock

**Affiliations:** 1 University of Birmingham and Centre for Clinical Haematology, Queen Elizabeth Hospital, Birmingham, UK

## 

In haematological malignancies acquired abnormalities in chromatin structure are increasingly recognised as an important mechanism of leukemogenesis. Hypermethylation of promoter CpG islands results in the down-regulation of a range of tumor suppressor and pro-apoptotic genes which is plausibly linked with the acquisition of a neoplastic phenotype. Similarly, acquired changes in the acetylation and methylation status of histones caused by distinct leukemia fusion proteins result in transcriptional deregulation which contributes to the disease phenotype in patients with Acute Myeloid Leukaemia (AML) and myelodysplasia (MDS). It is therefore of great interest that pharmacological agents such as DNA methyltransferase inhibitors (DNMTI) and histone deacetylase inhibitors (HDI) with the capacity to reverse acquired chromatin abnormalities demonstrate significant clinical activity in patients with high risk MDS and AML. Indeed the activity of DNMTIs such as 5-azacitidine (AZA), when administered alone or in combination with an HDI, has led to their emergence as one of the most important recent therapeutic advances in the management of MDS and AML in the last two decades. It remains the case however that the molecular mechanism by which this new class of agents exerts an anti-tumor effect remains unknown. Critically the extent to which their clinical activity is dependent on re-induction of epigenetically silenced tumour suppressor or pro-apoptotic genes or whether their anti-tumor effect is mediated through an alternative mechanism remains unknown.

It is now clear that the acquired abnormalities in chromatin structure which characterise many malignancies also result in reduced expression of a number of candidate tumor antigens, including members of the cancer testis antigen family such as MAGE-A1 and MAGE-A3. Importantly there is convincing evidence, in solid tumors and haematological malignancies, that DNMTIs such as AZA and 5-aza-2’-deoxycytidine (decitabine) up-regulate the expression of epigenetically repressed putative tumor antigens thereby potentially increasing tumor immunogenicity. Furthermore AZA also increases the expression of HLA Class 1 and co-stimulatory molecules such asCD80 and CD86 on the surface of leukemic blasts. The observation that demethylating agents may modulate a tumor-specific immune response also suggest that epigenetic therapies might be used to enhance the clinical activity of both autologous and allogeneic immunotherapeutic strategies. Studies from have previously demonstrated that up-regulation of the minor histocompatibility antigen HA-1 on solid tumors by decitabine sensitises tumor cells to recognition by HA-1 specific cytotoxic T lymphocytes and there is a now compelling case to investigate further the role of epigenetic agents as a priming agent in settings such as peptide vaccination. Epigenetic therapies also hold promise as a potential mechanism of selectively augmenting an immunologically mediated graft-versus-leukaemia (GVL) effect after allogeneic stem cell transplantation. In principle the ability of DNMTI to up-regulate the expression of epigenetically silenced tumor antigens without concomitantly increasing the expression of minor histocompatibility antigens on skin, gut or liver offers the possibility of increasing a GVL effect without a concomitant risk of GVHD. In this context the recent demonstration that post-transplant AZA, albeit at lower doses, is well-tolerated and induces a CD8+ T cell response to candidate tumor antigens post-transplant is of great interest and supports the further examination of AZA as post-transplant therapy.

